# Evidence of Mitochondrial Dysfunction in Bacterial Chondronecrosis With Osteomyelitis–Affected Broilers

**DOI:** 10.3389/fvets.2021.640901

**Published:** 2021-02-01

**Authors:** Alison Ferver, Elizabeth Greene, Robert Wideman, Sami Dridi

**Affiliations:** Center of Excellence for Poultry Science, University of Arkansas, Fayetteville, AR, United States

**Keywords:** mitochondrial dysfunction, lameness, broilers, chondronecrosis, osteomyelitis, BCO

## Abstract

A leading cause of lameness in modern broilers is bacterial chondronecrosis with osteomyelitis (BCO). While it is known that the components of BCO are bacterial infection, necrosis, and inflammation, the mechanism behind BCO etiology is not yet fully understood. In numerous species, including chicken, mitochondrial dysfunction has been shown to have a role in the pathogenicity of numerous diseases. The mitochondria is a known target for intracellular bacterial infections, similar to that of common causative agents in BCO, as well as a known regulator of cellular metabolism, stress response, and certain types of cell death. This study aimed to determine the expression profile of genes involved in mitochondrial biogenesis, dynamics, and function. RNA was isolated form the tibias from BCO-affected and healthy broilers and used to measure target gene expression via real-time qPCR. Mitochondrial biogenesis factors PGC-1α and PGC-1β were both significantly upregulated in BCO along with mitochondrial fission factors OMA1, MTFR1, MTFP1, and MFF1 as well as cellular respiration-related genes FOXO3, FOXO4, and av-UCP. Conversely, genes involved in mitochondrial function, ANT, COXIV, and COX5A showed decreased mRNA levels in BCO-affected tibia. This study is the first to provide evidence of potential mitochondrial dysfunction in BCO bone and warrants further mechanistic investigation into how this dysfunction contributes to BCO etiology.

## Introduction

Bacterial chondronecrosis with osteomyelitis (BCO) is a prominent cause of lameness in the poultry industry which predominantly affects fast growing and large broilers. Lameness in modern broilers poses both an animal welfare and a production concern as it has been shown that higher incidence of lameness is associated with suboptimal environments leading to other associated economic and health concerns including condemnations postmortem and footpad dermatitis (1). In the case of BCO related lameness, points of high mechanical stress within the avian skeletal system include the proximal femur head are the most common areas for BCO to occur (2, 3). Histological changes in the proximal head of femurs affected by BCO show evidence of wound sites within the epiphyseal region of the growth plate with clear chondronecrosis. These sites often transect vasculature within the highly vascularized growth plate leading to decreased blood flow to surrounding areas and an entry point for any circulating, collagen-binding bacteria (2–4). Numerous opportunistic bacterial species have been identified as causative agents in BCO, the most common being *Staphylococcus aureus* (3, 4). It is unclear if wound sights develop before bacterial infection occurs, but once infected, chondronecrosis is observed at varying degrees. A study of the microbiome within chicken growth plates showed a decrease in the diversity of bacterial communities in BCO affected femurs compared to normal with *Staphylococcus* species predominating, indicating a shift in the make-up of the microbiome with tissue damage and inflammation (5). Research into mitigating BCO in large broilers has primarily centered around probiotic and feed supplementation during production. While some of these supplements show to be promising, their proposed mechanisms target the bacterial load or entry by either improving gut integrity or improving the overall immune system response without fully investing the etiology of bacterial lead necrosis at the site of infection (6, 7). While further investigation into management and nutritional strategies and preventions are valuable, an understanding of the underlying molecular mechanisms involved in BCO has yet to be elucidated and would provide greater insight into treatment and prevention from both a management and potentially genetic standpoint. Although the exact pathology of BCO is debated, the three major components of BCO are clear, bacterial infection coinciding with attrition and inflammation of the bone.

The mitochondria play a pivotal role in energy homeostasis, bone growth and remodeling, and host-cell response to bacterial infection (8). Mitochondrial dysfunction can be a disruption in mitochondrial biogenesis, ATP production/bioenergetics, mitochondrial fission and fusion pathways, or mitochondrial driven cellular responses to stimuli such as environmental stress, cellular damage, bacterial infection, and inflammation (8). In the case of intracellular bacterial infection, like that of *Staphylococcus aureus*, the mitochondria is not only a bacterial target, but also a mode of host-cell defense through accumulation of reactive oxygen species (ROS) (8, 9). Mitochondrial dysfunction has been reported in neurological degenerative diseases such as Alzheimer's and Parkinson's disease as well as metabolic diseases including type 2 diabetes and associated osteomyelitis (8, 10–12). For these reasons, we sought to investigate the state of mitochondria in BCO-affected bone of modern broilers. To that end, we determined the expression profile of key regulatory genes for mitochondrial biogenesis, dynamics, and function. Our results show, for the first time, evidence of dysregulation of genes involved in mitochondria biogenesis (PGC-1α and β), dynamics (OPA1 and MFN2), cellular respiration and antioxidant response (ANT, COX5a, COXIV, and av-UCP), supporting a status of mitochondrial dysfunction in BCO.

## Materials and Methods

### Collection of BCO and Normal Bone Samples

All animal experiments were approved by the University of Arkansas (Fayetteville, AR) Animal Care and Use Committee (protocol number 15043) and were in accordance with recommendations in NIH's Guide for the Care and Use of Laboratory Animals. The BCO model and healthy counterparts were conducted as previously described (3, 4). Cobb 500 males were the same animals used previously (13). Briefly, animals had *ad libitum* access to fresh water and feed (3.9 Mcal metabolizable energy kg^−1^and 180 g crude protein kg^−1^) while the experiment took place in the Poultry Environmental Research Laboratory at the University of Arkansas Poultry Research Farm. Ambient temperature was lowered gradually from 32 to 25°C by 21 days of age. A light cycle of 23 h light/1 h dark was maintained along with an ~20% relative humidity until 56 days of age. At the end of the 56 days, animals were weighed, humanely euthanized, and immediately necropsied to determine presence of subclinical lesions in the proximal heads of both the femora and tibiae. Bone was selected macroscopically based on previously reported scale (3, 4). Normal bone was considered free of any necrosis or lesion and BCO-affected bone consisted of bone that had either tibial head necrosis (THN) or severe tibial head necrosis (THNs). Proximal portions of bone, primarily consisting of the growth plate, from both affected and unaffected animals were snap frozen in liquid nitrogen and stored at −80°C for later analysis.

### Real-Time Quantitative PCR

From the normal and BCO-affected bone samples (*n* = 6), total RNA was isolated in accordance with the protocol of Carter et al. (14). Cellular RNA was isolated using Trizol reagent (Life Technologies, Carlsbad, CA), based on manufacturer's instructions. RNA concentrations were determined using Synergy HT multimode microplate reader and total RNA was reverse transcribed using qScript cDNA SuperMix (Quanta Biosciences, Gaithsburg, MD). Amplification was achieved using Power SYBRGreen Master Mix (Life Technologies, Carlsbad, CA) and real-time quantitative PCR (7500 Real Time System; Applied Biosystems, Foster City, CA). The sequences for oligonucleotide primers for *r18s, av-UCP, ANT, NRF1, Ski, FOXO1, PPAR*α*, PPAR*γ*, PGC-1*α*, PGC-1*β*, COXIV, COX5a, D-loop (mtDNA), SSBP1, TFAM, MFN1, MFN2, DNM1, OPA1, OMA1, MTFR1, MTFP1*, and *MFF1* were previously published (15). Additional primers used were *FOXO3* (forward, 5′-GCTCCCGGACAAATTCGA-3′; and reverse, 5′-TCGCCAAAATCGGTGACAA-3′), *FOXO4* (forward, 5′-CTGGGATACCGGGTCTTGAG-3′, and reverse, 5′-GGCTATCTGTCGATTTGAGTAATGAA-3′), *Keap1* (forward, 5′- CGCCATCTGTTACAACC−3′; and reverse, 5′- GCGTAGATCCCGTCGAT -3′).

Real-time quantitative PCR cycling conditions were 50°C for 2 min, 95°C for 10 min and 40 cycles of a two-step amplification (95°C for 15 s followed by 58°C for 1 min). The dissociation protocol from the sequence detection system was used for melting curve analysis to exclude potential contamination of non-specific PCR products. Negative controls that were used as templates contained no reverse transcription products. Relative expression of target genes was determined using the 2^−ΔΔCT^ method and healthy bone tissues were used as calibrators (16).

### Statistical Analysis

Data were analyzed by Student *t*-test using GraphPad version 7.03 (GraphPad Software, Inc., LaJolla, CA). Results are expressed as means ±SEM, with *P* < 0.05 set as statistically significant.

## Results

**Figure 1:**
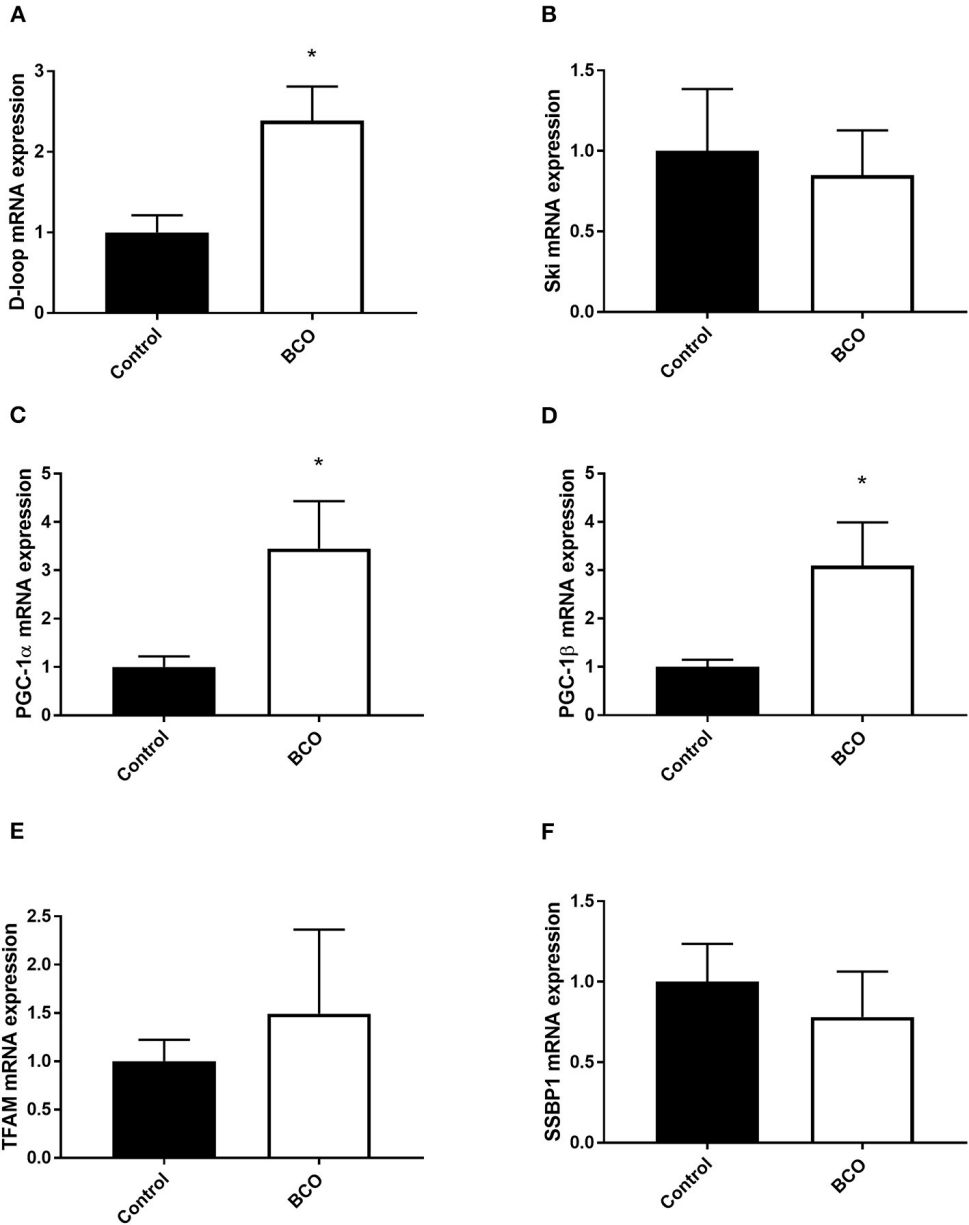
Mitochondrial biogenesis profile in BCO-affected birds. mRNA abundances of mitochondrial DNA, D-loop **(A)** as well as mitochondrial biogenesis regulators, Ski **(B)**, PGC-1α **(C)**, PGC-1β **(D)**, TFAM **(E)**, and SSBP1 **(F)** were determined by real-time qPCR. Data are presented as mean ± SEM (*n* = 6 birds/ group). *Denotes significant difference compared to the control group at *P* < 0.05. PGC-1, peroxisome proliferator-activated receptor gamma coactivator 1; SSBP1, single stranded DNA binding protein 1; TFAM, transcription factor A mitochondrial.

**Figure 2:**
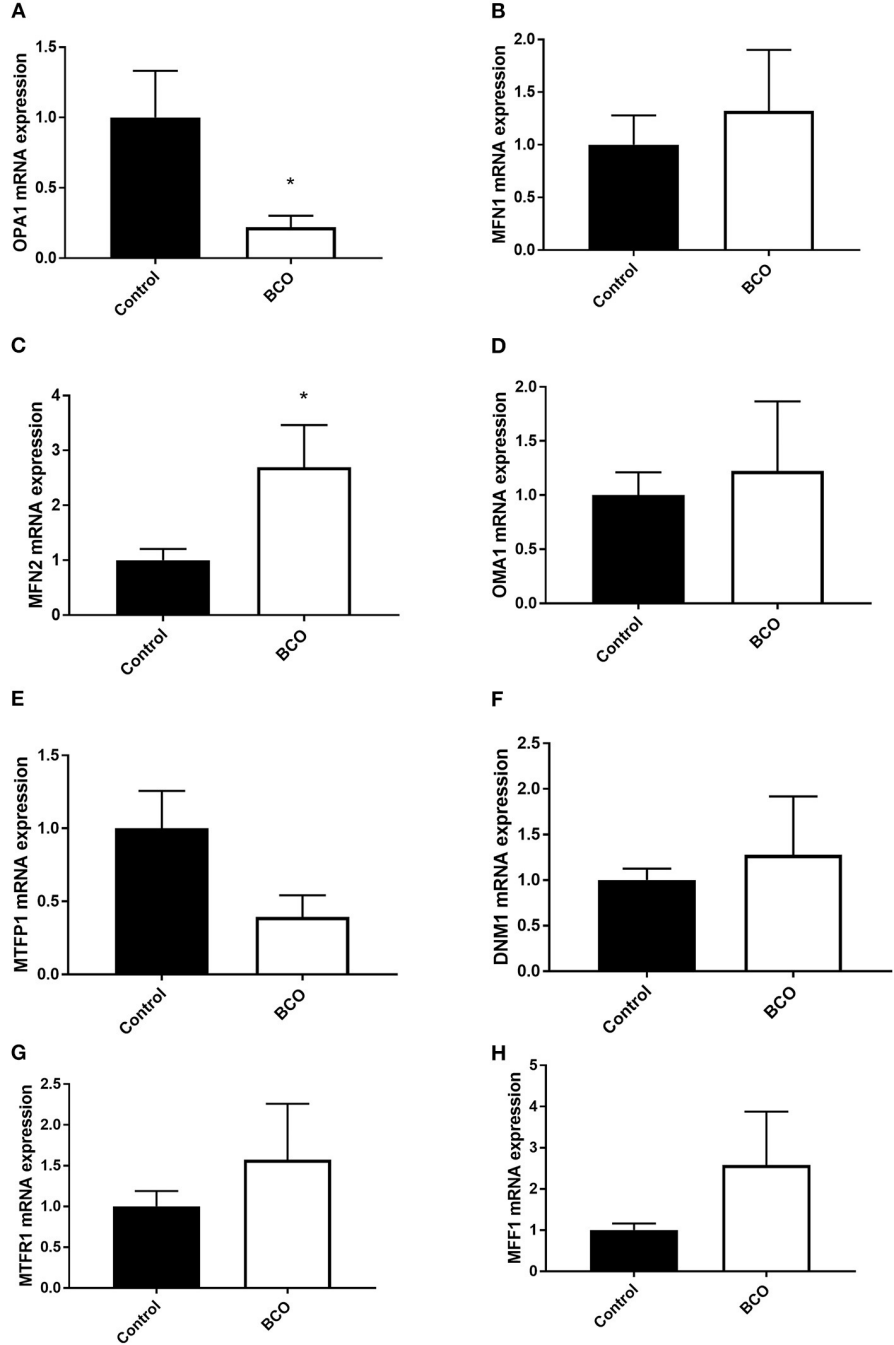
Mitochondrial dynamics profile in BCO-affected birds. The expression of genes regulating mitochondrial fusion, OPA1 **(A)**, MFN1 **(B)**, MFN2 **(C)**, and fission, OMA1 **(D)**, MTFP1 **(E)**, DNM1 **(F)**, MTFR1 **(G)**, and MFF1 **(H)** was determined by qPCR. Data are presented as mean ± SEM (*n* = 6 birds/ group). *Denotes significant difference compared to the control group at *P* < 0.05. DNM1, dynamin 1; MFF1, mitochondrial fission factor 1; MFN, mitofusin; MTFP1, mitochondrial fission process 1; MTFR1, mitochondrial fission regulator 1; OMA1, Overlapping With The m-AAA Protease 1; OPA1, optic atrophy protein 1.

Mitochondrial biogenesis-associated genes PGC-1α and PGC-1β were both significantly upregulated in BCO affected tissue as well as mitochondrial displacement loop (D-loop) (*P* < 0.05, Figures 1A,C,D). Mitochondrial transcription factor A (TFAM) and Ski followed a similar trend although not significantly upregulated (Figures 1B,E). Single-stranded DNA-binding protein 1 (SSBP1) mRNA expression was not significantly different between healthy control and BCO-affected group (Figure 1F). Mitochondrial dynamics indicated several differentially expressed genes including fusion related gene optic atrophy protein 1 (OPA1) expression being significantly down regulated (Figure 2A). Mitofusin 2 (MFN2), another fusion regulatory gene, was significantly upregulated, but MFN1 remained unchanged (*P* < 0.05, Figures 2B,C). The fission related genes, including overlapping with the M-AAA protease 1 homolog (OMA1), mitochondrial fission factor (MFF1) and mitochondrial fission regulator 1 (MTFR1), were not significantly upregulated in BCO-affected tissue (Figures 2D,G,H). Although mitochondrial fission process 1 (MTFP1) expression was decreased, it was also not statistically significant (Figure 2E). Dynamin 1 (DNM1) expression was not affected (Figure 2F).

**Figure 3:**
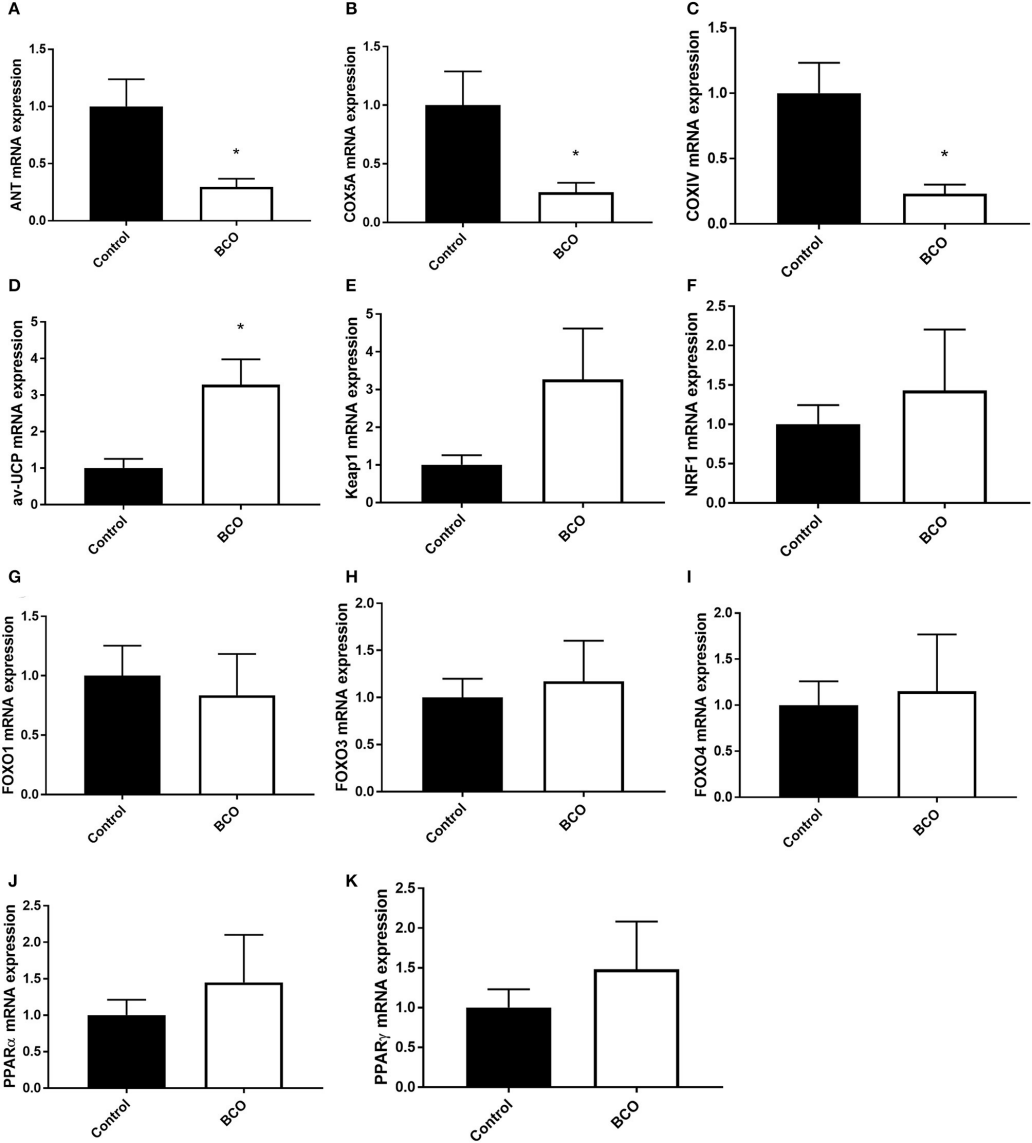
Mitochondrial function in BCO-affected birds. The expression of genes regulating mitochondrial function, ANT1 **(A)**, COX5A **(B)**, COXIV **(C)**, av-UCP **(D)**, Keap1 **(E)**, NRF1 **(F)**, FOXO1 **(G)**, FOXO3 **(H)**, FOXO4 **(I)**, PPARα **(J)**, and PPARγ **(K)** was measured by qPCR. Data are presented as mean ± SEM (*n* = 6 birds/group). *Denotes significant difference compared to the control group at *P* < 0.05. ANT1, adenine nucleotide translocase 1; av-UCP, avian uncoupling protein; COX, cytochrome c oxidase; FOXO, forkhead box protein; NRF1, nuclear respiratory factor 1; PPAR, peroxisome proliferator-activated receptor.

Gene expression was measured for regulators of mitochondrial function in ATP synthesis and overall respiratory activity (Figure 3). The gene for adenine nucleotide translocase (ANT) as well as cytochrome *c*-oxidase IV and 5A (COXIV, COX5A) were both significantly downregulated in BCO-affected tibia which coincided with a significant upregulation of avian uncoupling protein (av-UCP) (*P* < 0.05, Figures 3A–D). Although Kelch-like ECH-associated protein 1 (Keap1) and Forkhead box O3 and 4 transcription factors (FOXO3 and FOXO4) were not significantly upregulated in the case of BCO, they followed a similar positive trend (Figures 3E,H,I). Peroxisome proliferator activated receptors α and γ (PPARα and PPARγ) as well as nuclear factor erythroid 2-related factor 1 (NRF1) were non-significant, but upward trending (Figures 3F,J,K).

## Discussion

The role of mitochondria, its biogenesis, dynamics, and function, in disease states has become increasingly more poignant in the research community. In human disease, mitochondrial dysfunction, in the form of mtDNA mutations or disruption in overall mitochondrial biology, has been implicated in diabetes, Parkinson's disease, Alzheimer's disease, dementia, coronary artery disease, chronic fatigue syndrome, and ataxia (8–12, 17, 18). In broiler (meat-type) chickens, research has shown evidence of mitochondrial dysfunction related to susceptibility to ascites and to be influenced by orexin in broiler muscle (19, 20). Mitochondria are also direct targets for some bacterial infections including *Staphylococcus aureus*, one of the most common causative agents in BCO (9, 21). Mitochondrial dysfunction is believed to be associated with so many disease states due to its relation to apoptotic and inflammatory pathways (17). This study found evidence of mitochondrial dysfunction in the form of biogenesis regulation, fission or fractionation, and potential disruption of oxidative pathways.

Transcriptional control of mitochondrial biogenesis is heavily regulated via PGC-1α and PGC-1β (22). These coactivators both target transcription factors as well as act as targets for gene expression in mitochondrial biogenesis pathways (23). Extracellular signals such as metabolic changes and cell growth are known to modulate their expression, and upregulation of PGC-1α has been shown to coincide with increased mitochondrial biogenesis and respiration in inflammatory states (24, 25). In BCO-affected tissue, both PGC-1α and PGC-1β were significantly upregulated along with a non-significant but increased trend of TFAM. This increased expression suggests potential inflammatory or metabolic stressors activating mitochondrial biogenesis to offset deleterious effects of these states. D-Loop, a measurement of mtDNA, was also significantly upregulated, indicating an increase in mitochondrial DNA in BCO tissue (26). Although the state and quality of the mtDNA is not known, increased expression of key co-activators, along with increased mtDNA, could point to increased mitochondrial biogenesis in the presence of BCO. Since BCO is associated with bacterial infection, its inflammatory response could be the cause in increased need for mitochondria as ROS accumulation and metabolic shifts occur. SSBP1 is a housekeeping gene involved also in mitochondrial biogenesis and it has been shown to regulate mitochondrial mass in cancer states (27). SSBP1 expression was not significantly altered in BCO compared to healthy tibia suggesting that SSBP1 is potentially involved in the maintenance of genome stability rather than mitochondrial biogenesis in BCO pathogenesis.

Mitochondria are dynamic organelles with their shape and size shifting in response to numerous stimuli to maintain homeostasis or alter cellular energy states (28). Mitochondrial fusion is a state in which separate mitochondrial organelles fuse into a larger network and is often seen during stress conditions as it has the capacity to partially mix damaged mitochondria for complementation (28, 29). OPA1 is a key component of mitochondrial fusion found in the inner mitochondrial membrane and its knock down has resulted in blocked mitochondrial fusion and cellular defects including decreased cellular respiration and heterogeneity of mitochondrial membrane (29). In an ascites susceptible selected line of broilers, OPA1 expression has been shown to decrease under ascites conditions in the lungs, thigh, and breast compared to an ascites resistant selected line (19). In BCO, significant decrease in OPA1 mRNA expression was observed although MFN2 was upregulated. Mitofusins (MFN1 and MFN2) are found on the outer mitochondrial membrane and are necessary for mitochondrial fusion. However, without OPA1, the inner membrane is unable to fuse and the membrane potential can be implicated (29). This demonstrates that there exists a potential pathway of mitochondrial dysfunction via interruption of inner membrane fusion through OPA1 dysregulation in BCO. This dysregulation could be due to a shift in cell death pathways in which fracturing of the mitochondria is a key component, or it could be the result of mitophagy as dysfunctional mitochondria are processed by the cells via fractionation (30). The decrease in OPA1 expression was coupled with a trending increase expression of OMA1, a regulator of mitochondrial fission via “cutting” OPA1 at specific sites to render it inactive (31, 32). Mitochondrial fission enables the removal of damaged mitochondria through fragmentation when complementation via fusion is no longer possible, but also facilitates apoptosis in response to high levels of cellular stress (32). Although none were statistically significant, all fission gene expression followed a positive trend and analysis of protein expression could further elucidate the potential role of these genes. This increased expression of fission related genes further supports a cellular shift toward fission of mitochondria rather than fusion and suggests a potential state of mitochondrial turn-over in response to the high cellular stress during BCO. MTFR1 overexpression has been shown to promote mitochondrial fission (33). MTFP1 overexpression has been linked to increased ROS production and it has been shown to aid in mitochondrial recruitment of DNM1 along with MFF1 (34–37). These results show a decreased expression of a significant inner membrane fission factor OPA1 and point to potential fission/fragmentation of mitochondrial networks in BCO. Further research is needed to understand the potential impacts of this fission favored state in relation to BCO pathogenicity and whether it is a response to bacterial infection or a result of cellular removal and repair of necrotic tissue (37).

The main function of the mitochondria is to produce ATP for the cell. This is done through oxidative phosphorylation via the electron transport chain. Mitochondrial membrane potential as well as function cytochrome-c complexes are necessary for ATP production (38–41). Key proteins in this process, including ANT, COXIV, and COX5A, were significantly down regulated in BCO affected tissue. Conversely, av-UCP, an uncoupler of ATP production via movement of hydrogen ions across the mitochondrial inner membrane without ATP production, was significantly upregulated in BCO (42, 43). Av-UCP has been shown to be upregulated by pro-inflammatory cytokines such as interleukin 6 (IL-6) and tumor necrosis factor α (TNFα) via the transcription factors PGC-1α and PPARs (42). This imbalance of gene expression when compared to unaffected tissue points to potential dysfunction in ATP production in BCO via uncoupling of ATP production a potential loss of respiratory machinery and transporters such as COXIV and ANT. Beyond the machinery directly needed for ATP production, increased FOXO3 expression, although not significant, indicates possible reduction of respiratory activity through its repression of nuclear encoding mitochondrial genes (44). FOXO4 is a known activator of antioxidant genes, suggesting potential increased ROS as cellular respiration is impaired (45). PPARα and PPARγ were slightly upregulated, however not significant. PPARs are involved in mitochondrial function and have been implicated in diabetes-related mitochondrial dysfunction (46, 47). Overall, mitochondrial ATP production could be affected in BCO via increased av-UCP as an inflammatory response and decreased expression of mitochondrial transport proteins. This decreased in genes needed for proper mitochondrial ATP production could also be the result of mitochondrial turnover, as indicative of the fission pathways being activated in BCO. Whether a cause for or result of mitochondrial turnover, gene expression suggests dysregulation of ATP production pathways in BCO affected tissue.

Taken together, these results are the first to implicate potential mitochondrial dysfunction with BCO in modern broilers. Based on the expression of key mitochondrial related genes in BCO affected bone, upregulation of mitochondrial biogenesis, a known stress response, as well as mitochondrial fission pathways indicate latent mitochondrial turn over or fractionation coupled with disruption of ATP production machinery and transporters. More research is needed in order to elucidate the relationship between mRNA and protein expression in BCO as well as potential varying gene expressions in the severity of lesions or progression of BCO.

## Data Availability Statement

The original contributions presented in the study are included in the article/supplementary material, further inquiries can be directed to the corresponding author/s.

## Ethics Statement

All animal experiments were approved by the University of Arkansas (Fayetteville, AR) Animal Care and Use Committee (protocol number 15043) and were in accordance with recommendations in NIH's Guide for the Care and Use of Laboratory Animals.

## Author Contributions

SD design the study. AF and EG analyze the data. SD wrote the manuscript. RW, AF, and EG edit the manuscript. All authors contributed to the article and approved the submitted version.

## Conflict of Interest

The authors declare that the research was conducted in the absence of any commercial or financial relationships that could be construed as a potential conflict of interest.

## Abbreviations

ANT: adenine nucleotide translocase
ATP: adenosine triphosphate
av-UCP: avian uncoupling protein
BCO: bacterial chondronecrosis with osteomyelitis
cDNA: complementary deoxyribonucleic acid
COX: mitochondrial cytochrome C oxidase
DNM1: dynamin 1
FOXO: forkhead box O 4
Keap1: Kelch-like ECH-associated protein 1
MFF1: mitochondrial fission factor 1
MFN: mitofusin
mtDNA: mitochondrial DNA
MTFP1: mitochondrial fission process 1
MTFR1: mitochondrial fission regulator 1
NRF1: nuclear respiratory factor 1
OMA1: overlapping with the M-AAA protease 1 homolog
OPA1: optic atrophy protein 1
qPCR: quantitative polymerase chain reaction
RNA: ribonucleic acid
ROS: reactive oxygen species
SEM: standard error of the mean
SSBP1: single stranded DNA-binding protein 1
THN/THNs, tibial head necrosis/tibial head necrosis: severe
TFAM: mitochondrial transcription factor A.
